# Association between laboratory-based frailty index and unfavorable treatment outcomes in older adults with drug-susceptible pulmonary tuberculosis in the Republic of Korea: a retrospective cohort study

**DOI:** 10.3389/fpubh.2026.1873438

**Published:** 2026-07-10

**Authors:** Tae Eun Park, Hyun Ji Woo, Yeong Hun Choe, Min-Gul Kim

**Affiliations:** 1 Department of Pharmacology, Graduate School, Jeonbuk National University, Jeonju, Republic of Korea; 2 College of Pharmacy, Woosuk University, Wanju, Republic of Korea; 3 Clinical Trial Center, Jeonbuk National University Hospital, Jeonju, Republic of Korea; 4 Nanum Space Co. Ltd, Jeonju, Republic of Korea; 5 Department of Internal Medicine, Jeonbuk National University Hospital, Jeonju, Republic of Korea; 6 Research Institute of Clinical Medicine of Jeonbuk National University-Biomedical Research Institute of Jeonbuk National University Hospital, Jeonju, Republic of Korea; 7 Center for Clinical Pharmacology, Jeonbuk National University Hospital, Jeonju, Republic of Korea; 8 Department of Pharmacology, School of Medicine, Jeonbuk National University, Jeonju, Republic of Korea

**Keywords:** drug-susceptible pulmonary tuberculosis, frailty index, older adults, retrospective cohort study, treatment outcome

## Abstract

**Introduction:**

Older adults with drug-susceptible pulmonary tuberculosis (DS-PTB) face disproportionately poor treatment outcomes, but practical tools for risk stratification around the time of treatment initiation remain limited.

**Methods:**

This retrospective cohort study evaluated the association between the laboratory-based frailty index (FI-Lab) and unfavorable treatment outcomes in adults aged ≥65 years with DS-PTB, who were treated between January 1, 2015, and December 31, 2024, at a tertiary academic medical center in the Republic of Korea. The FI-Lab score was derived from routine blood tests collected within 60 days before to 7 days after treatment initiation, and the primary outcome was a composite of death during treatment, treatment failure, or treatment discontinuation.

**Results:**

Among 627 patients, 119 (19%) experienced an unfavorable outcome. Those in the highest FI-Lab quartile had greater odds of an unfavorable outcome than those in the lowest (odds ratio 3.42, 95% CI 1.75–6.69; *p* < 0.001; *p*-for trend = 0.0002), after adjustment for age, sex, comorbidity burden, treatment delay, baseline medications, cavitary disease, and bacteriological status. Results were consistent across seven pre-specified sensitivity analyses and in the Cox proportional hazards model (hazard ratio 3.28, 95% CI 1.60–6.73; *p* = 0.001).

**Discussion:**

Higher FI-Lab scores were independently associated with unfavorable treatment outcomes in older adults with DS-PTB. Derived from routinely available blood tests, FI-Lab may offer a practical approach to frailty quantification around the time of treatment initiation, and prospective studies are needed to evaluate whether frailty-targeted interventions improve outcomes.

## Introduction

Tuberculosis (TB) is a major global health challenge, and an estimated 10.7 million incident cases and 1.23 million deaths were reported in 2024 (1). Among those affected, adults aged ≥65 years have lower treatment completion rates and worse outcomes than younger adults (2, 3). They are particularly vulnerable because of age-related immunodeficiency, chronic comorbidities, and adverse drug events, including drug–drug interactions (2–4). These challenges are further complicated by frailty, which may present with malnourishment, falls, incontinence, and cognitive disorders. However, it is often under-recognized as the cause of suboptimal TB treatment outcomes in older adults.

In the Republic of Korea, TB incidence has declined over the past two decades from 100.8 per 100,000 population in 2011 to 33.5 per 100,000 in 2025 (5). However, the median age of patients rose from 51 years in 2011 to 68 years in 2022, with adults aged ≥65 years now accounting for 62.5% of all TB cases nationally (5, 6). Treatment outcomes in this age group are worse than in younger adults. In 2024, the treatment success rate was 72.4% for patients ≥65 years, compared with 90.8% for those <65 years, and the mortality during treatment reached 24.2% in the older group (5). Nationwide linked cohort analyses further showed that mortality during drug-susceptible pulmonary TB (DS-PTB) increased from 5.8% in 2011 to 15.3% in 2021, a rise closely associated with the aging of the TB population. More than half of these deaths were attributable to non-TB causes such as pneumonia and malignancy (6).

Frailty is a decline in physiological reserve that affects multiple organ systems due to aging. It impairs the ability to cope with stressors and increases the risk of morbidity and mortality (7). A meta-analysis across 62 countries estimated frailty prevalence at 12% by physical frailty measures and 24% by the frailty index (8). Frailty independently predicts adverse outcomes in serious infections. In older adults with pneumonia, a meta-analysis reported a frailty prevalence of 49% and two to three times greater odds of mortality among frail patients (9). In critically ill patients with sepsis, frailty was associated with increased one-year mortality after adjustment for age, comorbidities, and disease severity (10). These findings suggest that frailty predicts clinical outcomes across infection types independently of disease severity.

Two models have been proposed to measure frailty: the frailty phenotype, which defines frailty as a clinical syndrome requiring at least three of five criteria (weakness, slowness, exhaustion, low physical activity, and unintentional weight loss), and the frailty index, which quantifies frailty as the accumulation of deficits identified through geriatric assessment (7). Howlett and colleagues (11) developed a frailty index from laboratory data (FI-Lab) that does not require clinical assessments or questionnaires. In older adults, FI-Lab predicted all-cause mortality and unplanned hospitalization and correlated with clinical frailty ratings. A systematic review and meta-analysis confirmed its utility across community and hospital settings, supporting its applicability where formal functional assessment may not be feasible (12).

Currently, there is limited evidence on the use of FI-Lab in TB populations. Previous studies have assessed frailty based on comprehensive clinical evaluation tools and questionnaires. Li and colleagues (13) used four clinical assessment tools (the Tilburg Frailty Indicator, Social Support Scale, Barthel Index, and Geriatric Depression Scale) comprising 50 items that require direct patient evaluation in older adults with pulmonary TB. However, it did not examine the association between frailty and treatment outcomes. Sun and colleagues (14) developed a frailty index that identified 53 health deficits across seven dimensions, including cognitive function, activities of daily living, depressive symptoms, comorbidities, healthcare utilization, physical function, and laboratory results. It was associated with increased all-cause mortality in older adults with latent TB infection. The methods used by previous studies required dedicated clinical assessment beyond standard care. Without a practical frailty measure, identifying older TB patients at high risk of poor outcomes remains difficult. Therefore, we evaluated whether higher FI-Lab scores were independently associated with unfavorable treatment outcomes in older adults with DS-PTB.

## Materials and methods

### Study design and setting

This retrospective cohort study was conducted at Jeonbuk National University Hospital, Jeonju, Republic of Korea. We used a de-identified institutional dataset created on December 5, 2025, for a separate study, derived from the electronic health record comprising longitudinal data on diagnoses (ICD-10 codes), prescription records, laboratory and imaging results, sociodemographic data, and date of death. We identified an initial cohort of outpatients and inpatients with a pulmonary TB diagnosis (ICD-10 A15* or A16*) between January 1, 2015, and December 31, 2024. The study was approved by the Institutional Review Board of Jeonbuk National University Hospital (approval no. 2026–03-059), and the requirement for informed consent was waived because of the retrospective design and use of de-identified data.

### Eligibility criteria

Patients were eligible for inclusion if they: (i) were aged ≥65 years at the time of treatment initiation; (ii) initiated anti-TB therapy with at least three agents, including isoniazid and rifampicin, within 14 days of a pulmonary TB diagnostic code entry; and (iii) had a minimum of 180 days of follow-up available from the index date (Supplementary material). The earliest prescription date satisfying criterion (ii) was defined as the index date.

Patients were excluded if they: (i) had evidence of drug-resistant TB (receipt of any second-line anti-TB agent or rifampicin resistance on Xpert MTB/RIF assay); (ii) had evidence of non-tuberculous mycobacterial (NTM) disease (ICD-10 A31*; macrolide therapy ≥56 days; or NTM culture positivity); (iii) had fewer than 16 of 23 FI-Lab items within the baseline window (Supplementary material); (iv) had fewer than 30 days of concurrent isoniazid and rifampicin without a recorded death during the treatment episode, since these patients could not be classified into the pre-specified outcome categories; and (v) had evidence of prior TB treatment in a 180-day washout period before the index date. Patients whose 180-day washout period was before January 1, 2015, were retained in the primary analysis and excluded in a pre-specified sensitivity analysis.

### Laboratory-based frailty index

Twenty-three laboratory variables were collected from the dataset within a baseline window of 60 days before to 7 days after the index date. The dataset included reference ranges extracted from the electronic medical records with each measurement. If the institution revised its reference ranges during the study period, the range in effect at the time of testing was used (Supplementary material and Supplementary Table S1). Each laboratory test result was coded as a deficit if its value fell outside the reference range. When multiple results were available, the value nearest to the index date was used. The later result was selected when two values were equally distant from the index date. The FI-Lab score was calculated by dividing the number of deficits by the number of items measured. Patients were classified into quartiles (Q1 to Q4) based on the overall cohort distribution.

### Treatment outcomes

The primary outcome was unfavorable treatment outcome, which was a composite of: (i) death during treatment—all-cause death occurring during the treatment episode or within 30 days of its end; (ii) treatment failure—receipt of two or more anti-TB agents for >360 days without a treatment interruption of ≥60 days; and (iii) treatment discontinuation—premature cessation of anti-TB therapy defined as cumulative concurrent anti-TB agent days <150 days or a treatment gap of ≥60 days. Treatment episodes were defined as a series of consecutive prescriptions separated by <60 days. Cumulative concurrent anti-TB agent days (dual-drug days) were defined as days on which two or more anti-TB agents were prescribed concurrently, excluding days of monotherapy. Deaths during a treatment gap were classified as deaths during treatment rather than as discontinuation. Patients whose treatment was ongoing at the study end date of December 5, 2025, were excluded from the primary analysis (Supplementary material). Patients who died more than 30 days after their last anti-TB prescription did not meet the pre-specified criteria for either unfavorable outcome or treatment success and were not included in the outcome categorization.

Treatment success was categorized as standard success (150–270 dual-drug days) or extended success (271–360 dual-drug days), with no treatment gap of ≥60 days and no death during treatment. The lower threshold of 150 days was based on national guidelines, which allow treatment completion if at least 80% of the prescribed maintenance dose has been received. The upper threshold of 360 days aligns with the same guidelines: patients who received less than 80% of the prescribed maintenance dose and had a treatment interruption of less than 2 months should extend the maintenance phase to complete the remaining dose (15).

### Covariates

Demographic variables included age, sex, body mass index (BMI), insurance type (National Health Insurance vs. medical aid), and residence type (urban vs. rural, based on the patient’s registered address). Comorbidity burden was measured using the Rx-Risk Comorbidity Index based on prescriptions dispensed in the 365 days prior to the index date. Anti-TB agents were excluded to avoid capturing the index treatment episode as a comorbidity. The unweighted score was used in the primary analysis because the original weights reflect the contribution of each comorbidity to all-cause mortality rather than to TB treatment outcomes (16). Medications used 180 days before the index date were reviewed (corticosteroids, immunosuppressants, antidiabetic agents, antihypertensive agents, lipid-lowering agents, antiplatelet agents, anticoagulants, antipsychotics, benzodiazepines, and hypnotics/sedatives). Radiographic evidence of cavitary disease was determined from a chest radiograph or chest computed tomography (CT) performed within 30 days of the index date; CT took precedence if both were available. Reports were searched for the keywords, including “cavity,” “cavitary,” and “cavitation.” Acid-fast bacillus (AFB) smear results were obtained from sputum or bronchial washing specimens collected from 30 days before to 7 days after the index date. Smears were graded on a six-level scale (negative, trace, 1+, 2+, 3+, 4+). When multiple results were available, sputum specimens were prioritized over bronchial washing, and among specimens of the same type, the highest grade was selected. Smear positivity was defined as any grade above negative. Treatment delay was defined as the number of days from the first TB diagnostic code entry to the index date.

### Statistical analysis

Continuous variables are reported as medians with interquartile ranges (IQRs) and categorical variables as counts with percentages. Differences between the quartiles were assessed by the Kruskal–Wallis test and Chi-square test (or Fisher’s exact test when any expected cell count was <5) for continuous and categorical variables, respectively.

Logistic regression was performed in seven sequential models. Each model added one covariate block to the FI-Lab quartile, while Q1 served as a reference: Model 1, FI-Lab quartile alone; Model 2, demographics (age and sex); Model 3, clinical factors (BMI, Rx-Risk score, insurance type, and residence area); Model 4, treatment delay; Model 5, medications (corticosteroids, immunosuppressants, and antidiabetic agents); Model 6, cavitary disease; and Model 7, AFB smear positivity. In Model 5, corticosteroids, immunosuppressants, and antidiabetic agents were pre-specified for inclusion based on their biological plausibility as potential confounders of TB treatment outcomes (17, 18).

Multiple imputation by chained equations (MICE; 20 datasets) was used to handle missing data in key covariates, and it was the basis for the primary analysis across all seven models. Missing data were assumed to be missing at random (MAR) based on comparisons between patients with and without observed values (Supplementary Table S2) (19). Rubin’s rules were applied to the pooled model estimates and standard errors. A time-to-event analysis using Cox proportional hazards regression was fitted for Model 7, with time zero as the index date. Events were defined as in the primary analysis, and patients were censored at their last documented hospital contact or on December 5, 2025, whichever came first. The proportional hazards assumption was verified using Schoenfeld residual plots. To examine whether the results were robust to the competing event of death, a cause-specific hazard analysis was conducted.

Model discrimination was evaluated by the area under the receiver operating characteristic curve (AUC). To quantify the incremental value of adding FI-Lab to a base model comprising age, sex, and Rx-Risk score, the net reclassification improvement (NRI) and the integrated discrimination improvement (IDI) were calculated. Bootstrap validation with 1,000 resamples was used to correct AUC estimates for optimism. The Hosmer–Lemeshow test and decile calibration plots were used to assess calibration. Restricted cubic splines (RCS) were used to explore the dose–response relationship. Moreover, the following pre-specified sensitivity analyses were conducted: (i) alternative FI-Lab quartiles by Howlett and colleagues (<0.10, 0.10–0.22, 0.23–0.45, >0.45); (ii) restriction to patients with confirmed washout periods; (iii) extended treatment success reclassified as failure; (iv) a narrower baseline laboratory window (−30 to +7 days); (v) alternative Rx-Risk scoring methods and lookback windows; (vi) lower threshold of ≥180 days applied for treatment success; and (vii) a non-inflammatory FI-Lab excluding five inflammation-sensitive items (white blood cell count, neutrophil count, lymphocyte count, albumin, and C-reactive protein). Pre-specified subgroup analyses were conducted using Model 2 for the following variables: age, sex, corticosteroid use, immunosuppressant use, antidiabetic agent use, insurance type, baseline cavitary disease, baseline AFB smear status, cavitary disease at 2 months, and AFB smear status at 2 months. Firth penalized logistic regression was used in subgroups with too few outcome events for standard logistic regression.

All statistical analyses were conducted in Python 3.14.3. Analysis code for data preprocessing, cohort selection, FI-Lab calculation, and statistical modeling was developed with the assistance of Claude (version: claude.ai; model: Claude Sonnet 4.6; Anthropic, San Francisco, CA, United States), and all outputs were independently verified by the authors against the underlying data. A two-sided significance threshold of *α* = 0.05 was applied throughout. This study was reported in accordance with the Strengthening the Reporting of Observational Studies in Epidemiology (STROBE) guidelines (STROBE Checklist in Supplementary material).

## Results

### Cohort selection and FI-lab score distribution

**Figure 1:**
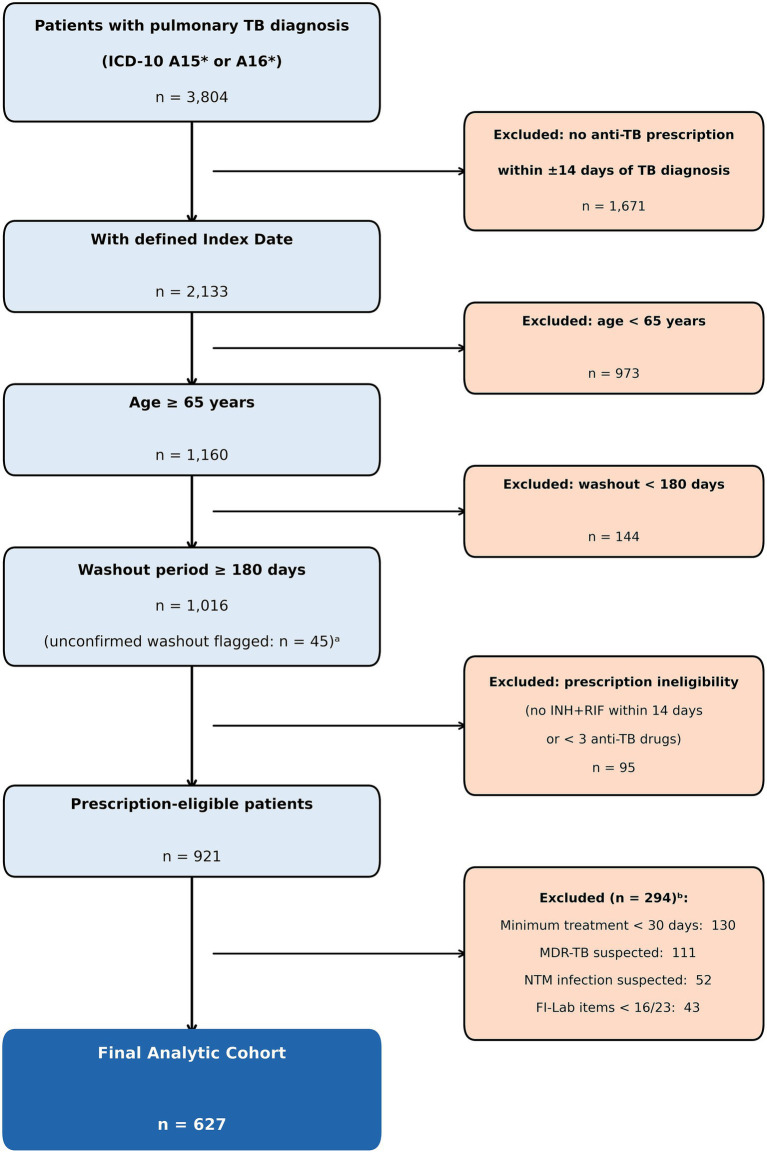
Patient selection flowchart. Flow diagram illustrating the stepwise selection of the study cohort. Starting from 3,804 patients with a tuberculosis diagnosis code (A15* or A16*), sequential exclusion criteria were applied. At each step, the number of patients excluded and the reasons for exclusion are shown. A total of 627 older adults met all inclusion criteria and were included in the final analytic cohort. ^a^Patients with index date before July 1, 2015: washout period could not be confirmed due to the data start date. They are included in the main analysis, but excluded in the sensitivity analysis (n = 45 in the final cohort). ^b^Exclusions are not mutually exclusive; patients meeting multiple criteria were counted once. FI-Lab, laboratory-based frailty index; INH, isoniazid; MDR, multidrug resistant; NTM, nontuberculous mycobacteria; RIF, rifampicin; TB, tuberculosis.

**Figure 2:**
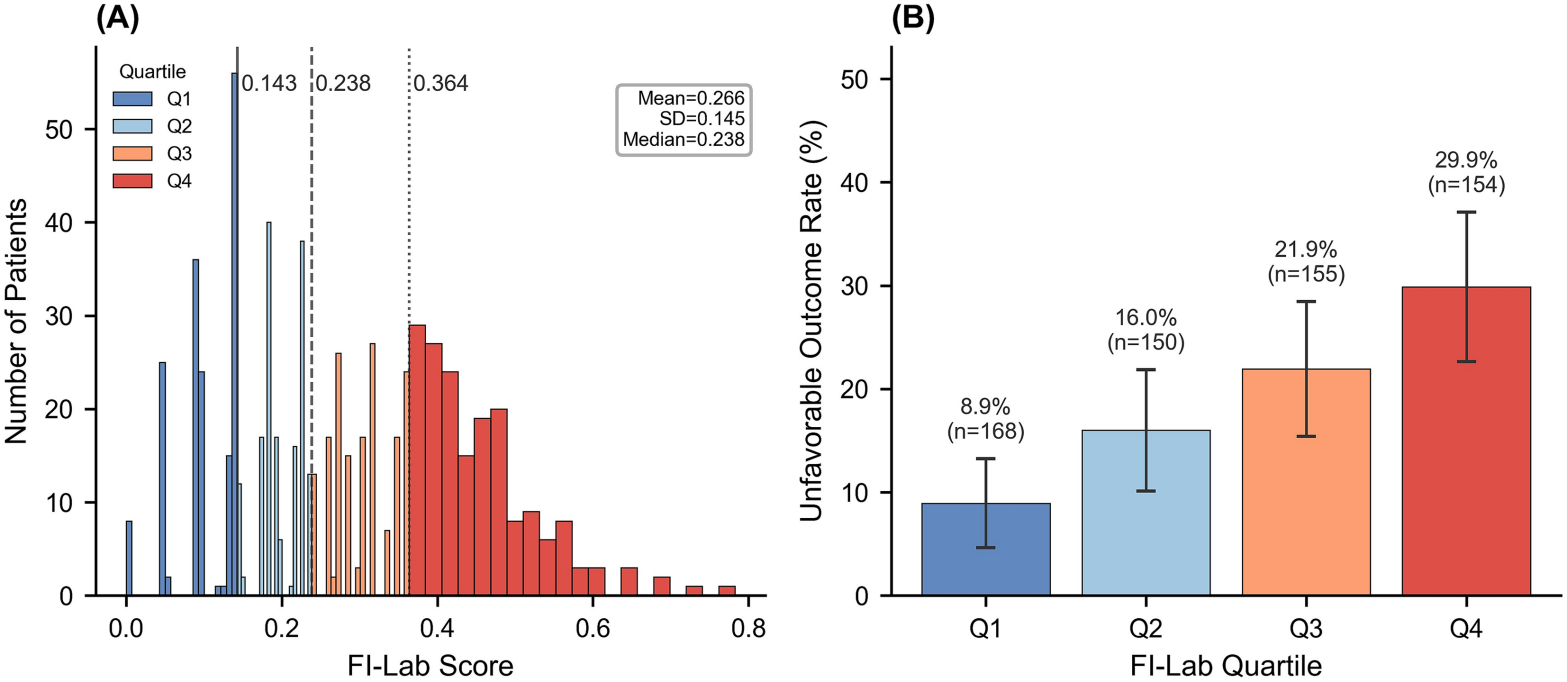
Distribution of FI-lab score and unfavorable outcome rate by quartile. **(A)** Histogram showing the distribution of laboratory-based frailty index (FI-Lab) scores among 627 patients. Vertical dashed lines indicate quartile boundaries. **(B)** Bar chart showing the proportion of patients with an unfavorable treatment outcome across FI-lab quartiles with 95% confidence intervals. A clear dose–response gradient is observed, as the highest quartile (Q4) shows a significantly higher rate of unfavorable outcomes than Q1.

Among 3,804 patients with pulmonary TB, 627 met eligibility criteria (Figure 1). The median FI-Lab score in our cohort was 0.238 (IQR 0.143–0.364; range 0–0.783). Quartile boundaries were the following: Q1 ≤ 0.143, Q2 > 0.143–0.238, Q3 > 0.238–0.364, and Q4 > 0.364 (Figure 2). Laboratory values comprising FI-Lab scores were collected near the index date (median: −1 day; IQR: −7 to +1 days), and 75% of those values were reported within the preceding 7 days.

### Baseline characteristics

Patients in higher FI-Lab quartiles were significantly older (median 75 years in Q1 vs. 79.5 years in Q4), more frequently hospitalized at the index date (38.7% in Q1 vs. 77.3% in Q4), had greater comorbidity burden (median Rx-Risk score 2 in Q1 vs. 4 in Q4), had a lower BMI (22.2 in Q1 vs. 20.9 kg/m^2^ in Q4), and had a shorter follow-up duration (median 834 in Q1 vs. 376 days in Q4) (Table 1). There were 73 (11.6%) and 83 (13.2%) patients whose BMI and baseline AFB smear results were missing, respectively. These variables were handled using MICE in the primary analysis (Supplementary Table S2).

**Table 1:** Baseline characteristics of the study cohort by FI-lab quartile.

Characteristic	Overall (*N* = 627)	Q1 (lowest) (*n* = 168) FI-lab score ≤0.143	Q2 (*n* = 150) 0.143–0.238	Q3 (*n* = 155) 0.238–0.364	Q4 (highest) (*n* = 154) FI-lab score >0.364	*p*-value
Age, years, median [IQR]	77.0 [72.0–82.0]	75.0 [70.0–81.0]	76.0 [71.0–81.0]	79.0 [73.0–82.5]	79.5 [74.0–83.0]	<0.001
Male sex, *n* (%)	341 (54.4%)	83 (49.4%)	83 (55.3%)	83 (53.5%)	92 (59.7%)	0.313
BMI, kg/m^2^, median [IQR] (missing *n* = 73)	21.7 [19.5–24.0]	22.2 [19.8–24.1]	21.8 [20.1–24.3]	21.8 [19.1–23.9]	20.9 [18.6–23.0]	0.011
Residence area, *n* (%)						0.834
Urban	286 (45.6%)	79 (47.0%)	65 (43.3%)	74 (47.7%)	68 (44.2%)	
Rural	341 (54.4%)	89 (53.0%)	85 (56.7%)	81 (52.3%)	86 (55.8%)	
Insurance type, *n* (%)						0.690
National health insurance	549 (87.6%)	150 (89.3%)	132 (88.0%)	136 (87.7%)	131 (85.1%)	
Medical aid	78 (12.4%)	18 (10.7%)	18 (12.0%)	19 (12.3%)	23 (14.9%)	
Care setting at index date, *n* (%)						<0.001
Outpatient	226 (36.0%)	101 (60.1%)	57 (38.0%)	45 (29.0%)	23 (14.9%)	
Inpatient	364 (58.1%)	65 (38.7%)	86 (57.3%)	94 (60.6%)	119 (77.3%)	
Emergency	37 (5.9%)	2 (1.2%)	7 (4.7%)	16 (10.3%)	12 (7.8%)	
FI-lab score, median [IQR]	0.238 [0.143–0.364]	0.095 [0.091–0.136]	0.191 [0.182–0.227]	0.304 [0.273–0.348]	0.455 [0.409–0.500]	
Follow-up duration, days, median [IQR]	597 [256–1,494]	834 [373–1,617]	765 [277–1,750]	533 [208–1,449]	376 [157–909]	<0.001
Treatment delay (diagnosis to first anti-TB agent), days, median [IQR]	0 [0–3]	0 [0-0]	0 [0–5]	0 [0–3]	0 [0–3]	
Rx-risk unweighted score, median [IQR]	3 [1–6]	2 [0–5]	2 [1–5]	3 [1–6]	4 [1–7]	<0.001
Corticosteroid use, *n* (%)	44 (7.0%)	5 (3.0%)	10 (6.7%)	14 (9.0%)	15 (9.7%)	0.074
Immunosuppressant use^a^, *n* (%)	7 (1.1%)	0 (0.0%)	2 (1.3%)	2 (1.3%)	3 (1.9%)	0.350
Antidiabetic agent use^a^, *n* (%)	70 (11.2%)	16 (9.5%)	13 (8.7%)	21 (13.5%)	20 (13.0%)	0.423
Cavitary disease on baseline imaging, *n* (%)	101 (16.1%)	22 (13.1%)	26 (17.3%)	22 (14.2%)	31 (20.1%)	0.322
Baseline AFB smear grade, median [IQR] (missing *n* = 83)	0 [0-1]	0 [0-0]	0 [0-1]	0 [0-1]	0 [0-1]	0.108

a ATC codes: immunosuppressants (L04A*), antidiabetic agents (A10A*, A10B*).

AFB, acid-fast bacilli; ATC, anatomical therapeutic chemical classification; BMI, body mass index; CT, computed tomography; CXR, chest X-ray; FI-Lab, laboratory-based frailty index; IQR, interquartile range; TB, tuberculosis.

### Treatment outcomes

Unfavorable treatment outcomes occurred in 119 patients (19%), and the rate increased progressively across FI-Lab quartiles from 8.9% in Q1 to 29.9% in Q4 (*p* < 0.001; Table 2). This gradient was primarily influenced by treatment discontinuation, which rose from 6.0% in Q1 to 22.1% in Q4. Treatment failure was rare (1.0%) and evenly distributed across quartiles.

**Table 2:** Treatment outcomes by FI-Lab quartile.

Treatment outcome	Overall^a^ (*N* = 627)	Q1 (lowest) (*n* = 168) FI-lab score ≤0.143	Q2 (*n* = 150) 0.143–0.238	Q3 (*n* = 155) 0.238–0.364	Q4 (highest) (*n* = 154) FI-lab score >0.364	*p*-value
Unfavorable treatment outcome	119 (19.0%)	15 (8.9%)	24 (16.0%)	34 (21.9%)	46 (29.9%)	<0.001
Death during treatment	24 (3.8%)	3 (1.8%)	3 (2.0%)	7 (4.5%)	11 (7.1%)	0.045
Treatment failure	6 (1.0%)	2 (1.2%)	2 (1.3%)	1 (0.6%)	1 (0.6%)	0.889
Treatment discontinuation	89 (14.2%)	10 (6.0%)	19 (12.7%)	26 (16.8%)	34 (22.1%)	<0.001
Treatment success	478 (76.2%)	149 (88.7%)	117 (78.0%)	113 (72.9%)	99 (64.3%)	<0.001
Standard (150–270 days)	424 (67.6%)	129 (76.8%)	102 (68.0%)	102 (65.8%)	91 (59.1%)	0.008
Extended (271–360 days)	54 (8.6%)	20 (11.9%)	15 (10.0%)	11 (7.1%)	8 (5.2%)	0.144

a Thirty patients (4.8%) who died more than 30 days after their last anti-TB prescription are not shown. Based on the pre-specified outcome definition, these deaths fell outside the “death during treatment” window, and these patients did not meet the criteria for treatment success.

FI-lab, laboratory-based frailty index.

**Figure 3:**
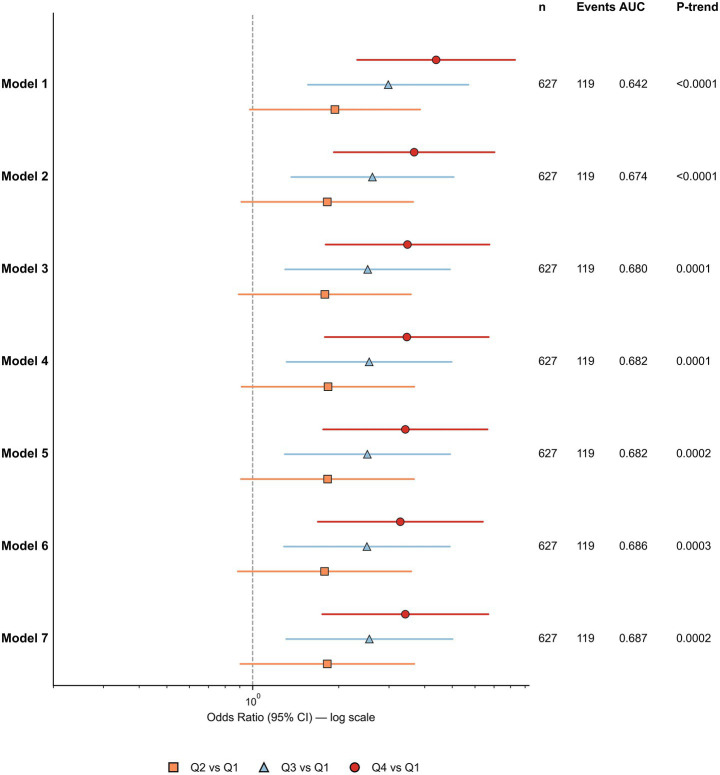
Association of FI-lab quartile with unfavorable treatment outcome across nested logistic regression models. Forest plot displaying odds ratios (ORs) and 95% confidence intervals (CIs) for FI-lab quartiles Q2, Q3, and Q4 versus Q1 (reference) across seven nested logistic regression models based on the MICE primary analysis. Model 1: FI-lab quartile only; Model 2: adjusted for age and sex; Model 3: additionally adjusted for BMI, Rx-Risk score, insurance type, and residence area; Model 4: additionally adjusted for treatment delay; Model 5: additionally adjusted for baseline medication use (corticosteroids, immunosuppressants, antidiabetic agents); Model 6: additionally adjusted for baseline cavitary disease; Model 7: fully adjusted (additionally adjusted for baseline AFB smear positivity). The rightmost columns report sample size, number of events, area under the receiver operating characteristic curve (AUC), and *p*-for trend across quartiles. AFB, acid-fast bacilli; BMI, body mass index; FI-Lab, laboratory-based frailty index; MICE, multiple imputation by chained equations.

**Figure 4:**
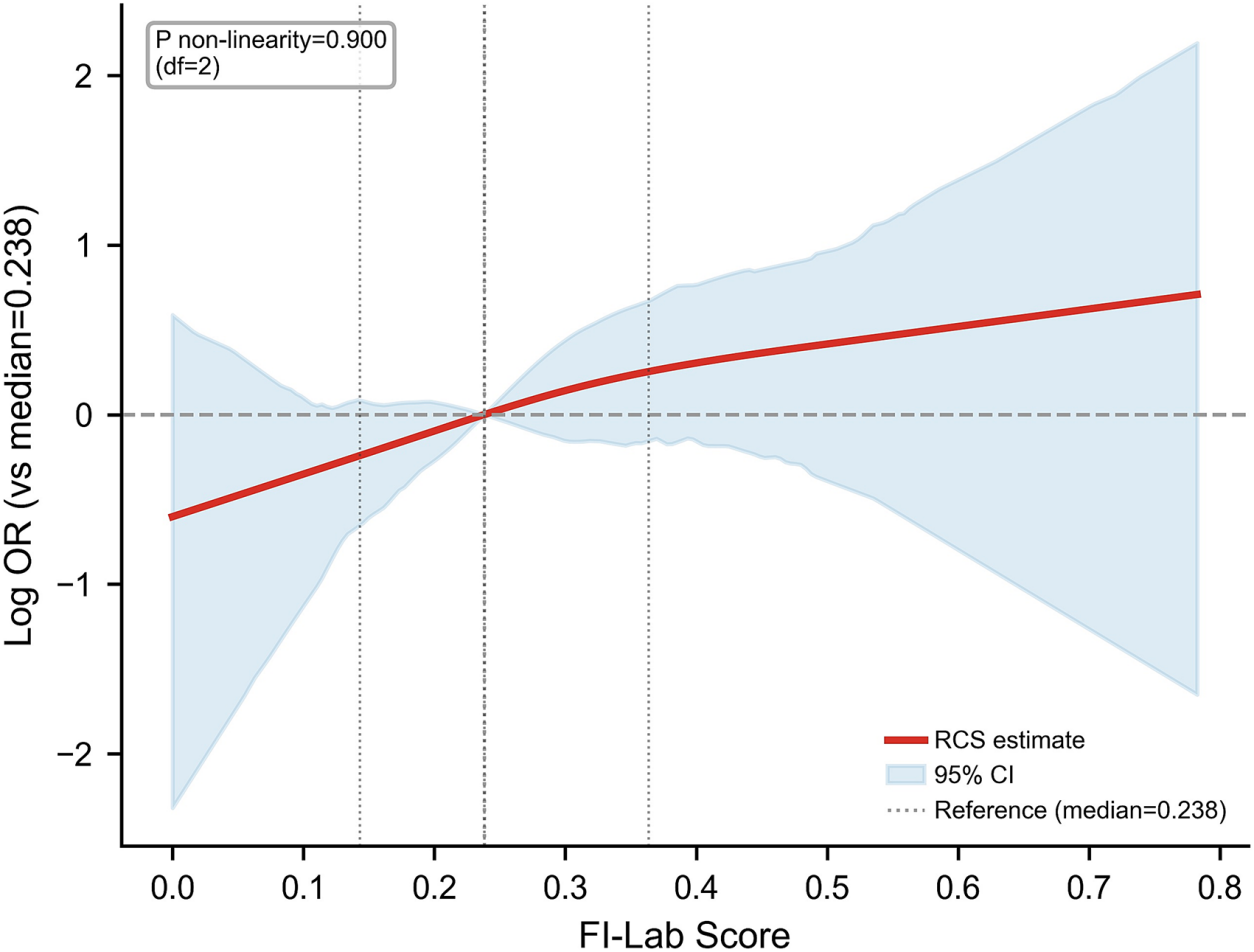
Restricted cubic spline analysis: FI-lab score and unfavorable treatment outcome. This restricted cubic spline plot shows the continuous association between the FI-lab score and the log odds ratio of an unfavorable treatment outcome. The model was fitted on complete case data (*n* = 554) and adjusted for age, sex, BMI, Rx-Risk score, insurance type, and residence area (Model 3 covariates). The continuous OR per 0.1-unit increment reported in the main text is from the MICE primary analysis (*n* = 627). BMI, body mass index; CI, confidence interval; d*f*, degree of freedom; FI-lab, laboratory-based frailty index; OR, odds ratio; RCS, restricted cubic spline.

In multivariable logistic regression, patients in the highest FI-Lab quartile (Q4) had 3.4 times the odds of unfavorable outcome compared with those in the lowest (Q1) after adjustment for age, sex, comorbidity burden, treatment delay, baseline medications, radiographic findings, and bacteriological status (MICE OR 3.42, 95% CI 1.75–6.69; *p* < 0.001; *p*-for trend = 0.0002; Model 7; Figure 3). The association attenuated when covariates were successively added for adjustments, but it remained statistically significant across all seven models. When FI-Lab was modeled as a continuous variable, each 0.1-unit increment was associated with a 26% increase in odds of unfavorable outcome (MICE OR 1.30, 95% CI 1.12–1.50; *p* < 0.001). Additionally, restricted cubic spline analysis supported a linear dose–response relationship across the observed range of FI-Lab scores (*p*-for non-linearity = 0.900; Figure 4). Results from the complete case analysis were consistent with the main findings (Supplementary Table S3). The Cox proportional hazards model was consistent with the primary analysis (Q4 vs. Q1: hazard ratio [HR] 3.28, 95% CI 1.60–6.73; *p* = 0.001; Supplementary Table S4). Schoenfeld residual tests showed that the effects of BMI (Models 3–7) and age (Models 2, 5, and 6) varied over time, whereas FI-Lab estimates remained stable across all models. In cause-specific hazard analysis, higher FI-Lab scores remained associated with treatment discontinuation after accounting for the competing event of death (*p*-for trend <0.001; Supplementary Table S5 and Supplementary Figure S1).

### Discrimination, sensitivity, and subgroup analyses

When FI-Lab was added to a base model of age, sex, and Rx-Risk score, the ability to identify patients at risk of unfavorable outcome improved (ΔAUC +0.047). The FI-Lab showed risk beyond what demographic factors and comorbidity burden alone could explain, and this was confirmed by bootstrap-derived NRI (0.342, 95% CI 0.140–0.523; *p* = 0.001) and IDI (0.026, 95% CI 0.013–0.038; *p* = 0.001; Supplementary Table S6). This augmented model was well-calibrated, as the predicted probabilities closely matched the observed event rates (Supplementary Figure S2). Moreover, it provided greater net benefit than the base model among patients with an estimated unfavorable outcome risk of 10–40% (Supplementary Figure S3).

The association between FI-Lab and unfavorable outcome was consistent across all seven pre-specified sensitivity analyses. Particularly, excluding five inflammation-sensitive items (white blood cell count, neutrophil count, lymphocyte count, albumin, and C-reactive protein) did not attenuate the association, indicating that the findings were not driven by acute inflammatory changes (Supplementary Table S7). No significant interaction was detected in any of the pre-specified patient subgroups, including age group, sex, medication use, insurance type, and disease status (all *p*-for interaction >0.10; Supplementary Table S8). However, point estimates in subgroups with few outcome events, such as corticosteroid use, immunosuppressant use, and disease status at 2 months, were imprecise and should be interpreted with caution.

## Discussion

Older adults now represent the majority of TB patients in the Republic of Korea, but their treatment outcomes remain worse than those of younger patients. This gap has not been adequately addressed, in part because frailty is not systematically assessed at the time of treatment initiation, despite evidence that it independently predicts adverse outcomes in serious infections (9, 10). Without a practical frailty measure, it is difficult to identify older adults at greatest risk before treatment begins. Our findings suggest that FI-Lab may help fill this gap.

This retrospective cohort study found that FI-Lab, which is a continuous frailty measure derived from routine blood tests, was independently associated with unfavorable treatment outcomes even after adjustment for comorbidity burden, treatment delay, baseline medications, radiographic findings, and bacteriological status. The association persisted across seven pre-specified sensitivity analyses and in Cox proportional hazards analysis. To our knowledge, this is the first study to demonstrate such an association in older adults with DS-PTB.

Frailty in older adults involves more than immune senescence against *M. tuberculosis* (20, 21). It reflects a broader decline in physiological and functional reserve, which makes it difficult to maintain a prolonged treatment course. In our cohort, treatment discontinuation was the predominant factor of unfavorable outcomes, and it increased steeply from the lowest to the highest FI-Lab quartiles. Because FI-Lab is derived from blood tests routinely collected at TB diagnosis, it may help identify patients who would benefit from closer follow-up, proactive outreach after missed visits, or early involvement of social support. Whether frailty-targeted interventions can improve TB treatment outcomes remains untested. Multicomponent interventions combining exercise and nutritional support have been shown to reduce frailty and improve physical function in older adults in other clinical contexts, but no randomized data exist in the TB population (22, 23). Prospective studies are needed to determine whether such interventions, delivered alongside standard anti-TB therapy, translate into improved treatment completion and survival.

Previously, Li and colleagues (13) assessed frailty in older adults with pulmonary TB but did not evaluate its association with treatment outcomes. Sun and colleagues (14) showed an association between frailty and mortality in latent TB infection, and their index required 53 items. Neither study evaluated the association between a laboratory-based frailty measure and treatment outcomes in active DS-PTB. Our study addresses this gap. The fact that FI-Lab is derived entirely from routinely collected blood tests during standard TB diagnostic workup distinguishes it from prior frailty tools used in TB research and may facilitate its integration into existing clinical workflows. Whether our findings generalize to primary care settings or populations with different TB burden and demographic profiles warrants evaluation in prospective, multicenter studies.

Unlike the Rx-Risk comorbidity score, FI-Lab showed an independent association with unfavorable outcomes in all adjusted models. This difference is clinically meaningful. The Rx-Risk index reflects disease burden based on prescriptions, whereas FI-Lab quantifies physiological deterioration through laboratory abnormalities. Patients with similar comorbidity burdens can differ in their underlying physiological reserve, and FI-Lab appears to capture this. The incremental discrimination analyses reinforce this interpretation: adding FI-Lab to a base model comprising age, sex, and Rx-Risk score improved the identification of patients at risk of unfavorable outcomes (Supplementary Table S6), suggesting that frailty reflects a risk dimension that comorbidity burden alone does not adequately represent.

However, it is worth considering whether FI-Lab reflects pre-existing frailty or acute TB-related laboratory changes. If the latter were the dominant contributor, FI-Lab would behave more as a disease severity marker than as a frailty measure. In our study, excluding five inflammation-sensitive items from the FI-Lab did not attenuate the association. The association also remained after adjustment for cavitary disease and AFB smear positivity, which are established indicators of TB disease burden. Nevertheless, the retrospective design cannot fully exclude a contribution from TB-induced changes that reverse with treatment, and prospective studies with serial measurements would help clarify this.

Our study (13) has several limitations. As a tertiary academic medical center, our institution likely enrolled a higher proportion of severely ill patients than those managed in primary care or community settings. If anything, having more severely ill patients in the cohort would have made the FI-Lab association harder to detect, so our estimates are likely conservative. Additionally, treatment records predating January 1, 2015, were inaccessible, and records from other institutions were unavailable. Therefore, some patients may have been misclassified as incident cases, even though they were actually receiving retreatment. However, a sensitivity analysis excluding patients with unconfirmed washout periods showed consistent results, suggesting that even if the misclassification was present, it did not affect the primary findings. Our study was restricted to adults aged ≥65 years; whether FI-Lab retains similar interpretability in younger patients, or whether age acts as an effect modifier across a broader age range, cannot be determined from the current data.

Fluoroquinolones were not used as an exclusion criterion for multidrug-resistant (MDR) TB because prescription records alone do not distinguish the difference between their use as a substitute for ethambutol due to adverse drug reactions and their use as part of an MDR-TB regimen. Additionally, isoniazid susceptibility testing results were unavailable because it was outsourced; therefore, isoniazid mono-resistant cases could not be excluded. However, fluoroquinolone prescriptions without other second-line TB agents were rare in our cohort, suggesting that the number of undetected resistant cases is likely small.

Deaths were counted only if they occurred at or were reported to our institution. This could have affected the death-during-treatment outcome. However, deaths unknown to our institution most likely would have occurred in frailer patients, making our estimates conservative instead of inflated. Additionally, the effects of BMI and age on the outcome changed over time, whereas the association between the FI-Lab and the outcome was stable throughout the observation period according to the Cox proportional hazards analysis. Finally, potential confounders, such as frailty trajectory, nutritional status, and functional status, were not accounted for in our study. Therefore, the observed association may partly reflect the influence of these unmeasured factors.

In conclusion, the FI-Lab, which quantifies frailty from routine blood tests, was independently associated with unfavorable treatment outcomes in older adults with DS-PTB. The dose–response relationship across FI-Lab quartiles was consistent in sensitivity analyses and time-to-event models. Together, our findings suggest that frailty reflects risk beyond what standard clinical and microbiological factors can explain. Whether targeting frailty along with anti-TB therapy can improve treatment outcome warrants prospective evaluation.

## Data Availability

The data analyzed in this study is subject to the following licenses/restrictions: The dataset used in this study was compiled from de-identified electronic medical records at Jeonbuk National University Hospital and cannot be shared publicly due to patient privacy regulations and institutional data governance policies. Requests to access these datasets should be directed to Min-Gul Kim, mgkim@jbnu.ac.kr.
