# Supplementary material for: Association between laboratory-based frailty index and unfavorable treatment outcomes in older adults with drug-susceptible pulmonary tuberculosis in the Republic of Korea: a retrospective cohort study
Source: Front Public Health. 2026 Jul 10;14:1873438. doi: 10.3389/fpubh.2026.1873438 (PMC13396249; doi:10.3389/fpubh.2026.1873438)
Supplement: Supplementary file 1 [file Data_Sheet_1.PDF]

Supplementary Information

Supplementary Methods 2

1. Frailty Index Based on Laboratory Tests (FI-Lab) 2

2. Treatment Outcome Classification 2

Supplementary Table S1: FI-Lab 23-Item Reference Ranges 3

Supplementary Table S2: Missing Data Analysis 4

Supplementary Table S3: Complete Case Logistic Regression 5

Supplementary Table S4: Cox Proportional Hazards Analysis 7

Supplementary Table S5: Competing Risks Analysis 9

Supplementary Table S6: Net Reclassification Improvement (NRI) and Integrated Discrimination Improvement (IDI)[a] 10

Supplementary Table S7: Sensitivity Analyses 11

Supplementary Table S8: Subgroup Analysis (FI-Lab Q4 vs Q1, Model 2[a]) 12

Supplementary Figure S1: Cumulative Incidence Functions – Competing Risks Analysis 13

Supplementary Figure S2: Calibration Plot 14

Supplementary Figure S3: Decision Curve Analysis – Net Benefit of FI-Lab-Augmented Model 15

STROBE Checklist – Cohort Study 16

References 18

Supplementary Methods

1. Frailty Index Based on Laboratory Tests (FI-Lab)

FI-Lab was constructed following the deficit accumulation model originally described by Howlett and colleagues (1). Twenty-three laboratory items were selected based on availability in the electronic medical records (EMR): hemoglobin (Hb), platelet count, white blood cell count (WBC), neutrophil count, lymphocyte count, monocyte count, red cell distribution width (RDW), mean corpuscular volume (MCV), AST, ALT, albumin, total protein, total bilirubin, glucose, sodium, potassium, phosphorus, blood urea nitrogen (BUN), creatinine, C-reactive protein (CRP), total calcium, HbA1c, and chloride.

Patients with fewer than 16 of the 23 measured items were excluded based on the 70% minimum threshold described in previous studies (1,2). Several items had their reference ranges updated by the laboratory during the study period: AST, ALT, Creatinine, Sodium, Potassium, Chloride, Glucose, Albumin, Total Calcium, and CRP were updated in 2022; the upper limit of normal for hemoglobin in males was updated in 2021. The complete list of items, stratification type, and applicable reference ranges is presented in Supplementary Table 1.

2. Treatment Outcome Classification

Treatment episodes were identified from prescription records. A gap was defined as the interval between the end of one prescription (start date + duration days – 1) and the start date of the next; episodes were separated when this interval exceeded 60 days.

Follow-up duration was measured from the index date to the earliest of four dates: (i) the date of in-hospital death; (ii) the end date of the last anti-TB prescription; (iii) the date of the last TB-related healthcare contact (defined as any visit or diagnostic test recorded with a TB diagnostic code); or (iv) the date of the last recorded activity in the hospital system regardless of the diagnosis. The follow-up date must be no later than December 5, 2025.

Supplementary Table S1: FI-Lab 23-Item Reference Ranges

#	FI-Lab item (code)	Reference range	Unit	Notes
1	Hemoglobin (L200203)	M: 13.0-18.0<br>F: 12.0-16.0	g/dl	Male upper limit updated to 16.5 in 2021
2	Platelet count (L200209)	130.0-450.0	$x10^3/\mu L$	
3	White blood cell count (L200201)	4.8-10.8	$x10^3/\mu L$	
4	Neutrophil count (L200318)	1.8-7.8	$x10^3/\mu L$	
5	Lymphocyte count (L200316)	1.0-4.8	$x10^3/\mu L$	
6	Monocyte count (L200317)	0.0-0.8	$x10^3/\mu L$	
7	Red cell distribution width (L200208)	11.5-14.5	%	
8	Mean corpuscular volume (L200205)	M: 80.0-94.0<br>F: 81.0-99.0	fL	
9	Aspartate aminotransferase (L3015)	12.0-33.0	IU/L	Updated to 0.0-40.0 in 2022
10	Alanine aminotransferase (L3016)	5.0-35.0	IU/L	Updated to 0.0-41.0 in 2022
11	Albumin (L3021)	3.5-5.3	g/dl	Updated to 3.5-5.2 in 2022
12	Total protein (L3020)	6.7-8.3	g/dL	Updated to 6.4-8.3 in 2022
13	Total bilirubin (L3018)	0.2-1.2	mg/dl	Updated to 0.0-1.2 in 2022
14	Fasting glucose (L3033)	74.0-106.0	mg/dl	Updated to 70.0-100.0 in 2022
15	Sodium (L3010)	135.0-150.0	mmol/L	Updated to 136.0-145.0 in 2022
16	Potassium (L3011)	3.5-5.5	mmol/L	Updated to 3.5-5.1 in 2022
17	Phosphorus (L3028)	2.5-4.5	mg/dl	
18	Blood urea nitrogen (L3024)	8.0-23.0	mg/dl	
19	Creatinine (L3025C)	0.7-1.7	mg/dl	Updated to 0.7-1.2 in 2022
20	C-reactive protein (L3052)	<5.0	mg/L	Updated to 0.0-5.0 in 2022
21	Total calcium (L3027)	8.4-10.2	mg/dl	Updated to 8.8-10.2 in 2022
22	Glycated hemoglobin (HbA1c) (L3034)	4.8-5.9	%	
23	Chloride (L3012)	91.0-110.0	mmol/L	Updated to 98.0-107.0 in 2022

Abbreviations: F, female; FI-Lab, laboratory-based frailty index; M, male

Supplementary Table S2: Missing Data Analysis

Variable	N total	N missing	% missing
FI-Lab score	627	0	0.0%
Age (continuous)	627	0	0.0%
Sex (female)	627	0	0.0%
BMI (kg/m²)	627	73	11.6%
Rx-Risk score (unweighted)	627	0	0.0%
Medical aid	627	0	0.0%
Residence area (rural)	627	0	0.0%
Treatment delay (days)	627	0	0.0%
Corticosteroid dose group	627	0	0.0%
Immunosuppressant use	627	0	0.0%
Antidiabetics use	627	0	0.0%
Baseline cavitary disease (CXR/CT)	627	1	0.2%
Baseline AFB smear positivity	627	83	13.2%
Unfavorable outcome (primary composite)	627	0	0.0%
Death during treatment	627	0	0.0%
Death after treatment	627	0	0.0%
Treatment discontinuation	627	0	0.0%

Abbreviations: AFB, acid-fast bacilli, BMI, body mass index; CT, computed tomography; CXR, chest X-ray; FI-Lab, laboratory-based frailty index

Missing Data Mechanism Assessment

Variable	N missing	% missing	Significant comparisons (n)	Conclusion
BMI	73	11.6%	5	MCAR[a] rejected → MAR assumed
Baseline cavitary disease	1	0.2%	0	Consistent with MCAR[a]
Baseline AFB smear positivity	83	13.2%	0	Consistent with MCAR[a]

[a] MCAR was assessed by comparing available vs. missing groups for each variable across seven analytic comparators (age, sex, FI-Lab score, poor outcome, death during treatment, medical aid, follow-up days) using Mann-Whitney U for continuous variables and Chi-square or Fisher's exact tests for categorical variables. "Significant comparisons (n)" denotes the number of these comparators differing at p<0.05. Patients missing BMI differed significantly from those with observed values on five of seven comparators (age, sex, FI-Lab score, unfavorable-outcome rate, and follow-up duration, all P<0.05), indicating that MCAR could not be assumed; MAR was addressed by MICE.

Abbreviations: AFB, acid-fast bacilli; BMI, body mass index; CT, computed tomography; CXR, chest X-ray; MAR, missing at random; MCAR, missing completely at random

Supplementary Table S3: Complete Case Logistic Regression

Model/Variable	OR (95% CI)	P value	P for trend	AUC
**Model 1: FI-Lab quartile only [n=627, events=119]**				
FI-Lab Q1 (reference)	Reference	-	<0.0001	0.641
FI-Lab Q2 vs Q1	1.94 (0.98-3.86)	0.058		
FI-Lab Q3 vs Q1	2.87 (1.49-5.50)	0.002		
FI-Lab Q4 vs Q1	4.34 (2.31-8.18)	<0.0001		
FI-Lab (per 0.1 unit, continuous)	1.39 (1.21-1.59)	<0.0001		
**Model 2: M1 + age, sex [n=627, events=119]**				
FI-Lab Q1 (reference)	Reference	-	<0.0001	0.672
FI-Lab Q2 vs Q1	1.81 (0.91-3.62)	0.093		
FI-Lab Q3 vs Q1	2.52 (1.30-4.87)	0.006		
FI-Lab Q4 vs Q1	3.63 (1.91-6.91)	0.0001		
Age (per year)	1.05 (1.02-1.08)	0.002		
Female sex	0.61 (0.40-0.94)	0.026		
FI-Lab (per 0.1 unit, continuous)	1.32 (1.15-1.52)	0.0001		
**Model 3: M2 + BMI, Rx-Risk, insurance, residence [n=554, events=92]**				
FI-Lab Q1 (reference)	Reference	-	0.017	0.665
FI-Lab Q2 vs Q1	1.67 (0.82-3.40)	0.157		
FI-Lab Q3 vs Q1	2.13 (1.06-4.29)	0.035		
FI-Lab Q4 vs Q1	2.37 (1.17-4.80)	0.017		
Age (per year)	1.04 (1.00-1.07)	0.036		
Female sex	0.56 (0.34-0.91)	0.020		
BMI (per kg/m²)	0.97 (0.91-1.04)	0.376		
Rx-Risk score (unweighted)	1.00 (0.93-1.06)	0.883		
Medical aid	1.81 (0.99-3.32)	0.056		
Rural residence	0.94 (0.59-1.50)	0.806		
FI-Lab (per 0.1 unit, continuous)	1.21 (1.03-1.43)	0.023		
**Model 4: M3 + treatment delay [n=554, events=92]**				
FI-Lab Q1 (reference)	Reference	-	0.020	0.671
FI-Lab Q2 vs Q1	1.74 (0.85-3.55)	0.129		
FI-Lab Q3 vs Q1	2.19 (1.09-4.43)	0.029		
FI-Lab Q4 vs Q1	2.35 (1.16-4.77)	0.018		
Age (per year)	1.04 (1.00-1.07)	0.041		
Female sex	0.55 (0.33-0.90)	0.018		
BMI (per kg/m²)	0.97 (0.90-1.04)	0.355		
Rx-Risk score (unweighted)	1.01 (0.95-1.08)	0.720		
Medical aid	1.78 (0.97-3.28)	0.064		
Rural residence	0.94 (0.59-1.50)	0.797		
Treatment delay (per 14 days)	0.57 (0.28-1.16)	0.121		
FI-Lab (per 0.1 unit, continuous)	1.20 (1.02-1.42)	0.027		
**Model 5: M4 + corticosteroids, immunosuppressants, antidiabetic agents [n=554, events=92]**				
FI-Lab Q1 (reference)	Reference	-	0.027	0.673
FI-Lab Q2 vs Q1	1.72 (0.84-3.51)	0.139		
FI-Lab Q3 vs Q1	2.14 (1.06-4.34)	0.035		
FI-Lab Q4 vs Q1	2.28 (1.12-4.64)	0.023		
Age (per year)	1.04 (1.00-1.08)	0.029		
Female sex	0.55 (0.33-0.90)	0.017		
BMI (per kg/m²)	0.97 (0.90-1.04)	0.353		
Rx-Risk score (unweighted)	1.00 (0.92-1.08)	0.944		
Medical ai	1.77 (0.96-3.27)	0.069		
Rural residence	0.95 (0.59-1.51)	0.816		
Treatment delay (per 14 days)	0.55 (0.27-1.14)	0.110		
Corticosteroid dose group	1.37 (0.86-2.19)	0.185		
Immunosuppressant use	1.06 (0.09-12.00)	0.964		
Antidiabetic agent use	0.97 (0.42-2.22)	0.934		
FI-Lab (per 0.1 unit, continuous)	1.20 (1.02-1.42)	0.032		

**Model 6: M5 + baseline cavitary disease [n=553, events=92]**				
FI-Lab Q1 (reference)	Reference	-	0.027	0.674
FI-Lab Q2 vs Q1	1.70 (0.83-3.47)	0.149		
FI-Lab Q3 vs Q1	2.14 (1.05-4.33)	0.035		
FI-Lab Q4 vs Q1	2.26 (1.11-4.61)	0.025		
Age (per year)	1.04 (1.00-1.08)	0.027		
Female sex	0.55 (0.33-0.90)	0.018		
BMI (per kg/m²)	0.97 (0.90-1.04)	0.385		
Rx-Risk score (unweighted)	1.00 (0.92-1.08)	0.969		
Medical aid	1.74 (0.94-3.23)	0.078		
Rural residence	0.96 (0.60-1.53)	0.852		
Treatment delay (per 14 days)	0.57 (0.27-1.17)	0.125		
Corticosteroid dose group	1.37 (0.86-2.19)	0.183		
Immunosuppressant use	1.06 (0.09-11.97)	0.960		
Antidiabetic agent use	0.95 (0.41-2.19)	0.908		
Baseline cavitary disease	1.21 (0.66-2.21)	0.542		
FI-Lab (per 0.1 unit, continuous)	1.20 (1.01-1.41)	0.034		
**Model 7: M6 + baseline AFB smear positivity [n=485, events=83]**				
FI-Lab Q1 (reference)	Reference	-	0.0075	0.690
FI-Lab Q2 vs Q1	1.82 (0.84-3.94)	0.126		
FI-Lab Q3 vs Q1	2.45 (1.15-5.22)	0.020		
FI-Lab Q4 vs Q1	2.84 (1.32-6.13)	0.008		
Age (per year)	1.04 (1.00-1.08)	0.036		
Female sex	0.52 (0.31-0.89)	0.016		
BMI (per kg/m²)	0.99 (0.92-1.07)	0.741		
Rx-Risk score (unweighted)	1.02 (0.94-1.10)	0.712		
Medical aid	1.82 (0.93-3.57)	0.080		
Rural residence	1.08 (0.65-1.78)	0.762		
Treatment delay (per 14 days)	0.45 (0.20-1.01)	0.054		
Corticosteroid dose group	1.23 (0.74-2.05)	0.421		
Immunosuppressant use	0.90 (0.08-10.45)	0.934		
Antidiabetic agent use	0.84 (0.34-2.05)	0.700		
Baseline cavitary disease	1.17 (0.61-2.27)	0.635		
Baseline AFB smear positivity	0.62 (0.34-1.15)	0.130		
FI-Lab (per 0.1 unit, continuous)	1.26 (1.06-1.51)	0.010		

Abbreviations: AFB, acid-fast bacilli, AUC, area under the receiver operating characteristic curve; BMI, body mass index; CI, confidence interval; FI-Lab, laboratory-based frailty index; OR, odds ratio

Supplementary Table S4: Cox Proportional Hazards Analysis

Model/Variable	HR (95% CI)	P value	P for trend	PH assumption[a]
**Model 1: FI-Lab quartile only [n=627, events=119]**				
FI-Lab Q1 (reference)	Reference	-	<0.0001	None
FI-Lab Q2 vs Q1	1.85 (0.97-3.53)	0.062		
FI-Lab Q3 vs Q1	2.88 (1.57-5.30)	0.0007		
FI-Lab Q4 vs Q1	4.24 (2.36-7.62)	<0.0001		
FI-Lab (per 0.1 unit, continuous)	1.37 (1.22-1.54)	<0.0001		
**Model 2: M1 + age, sex [n=627, events=119]**				
FI-Lab Q1 (reference)	Reference	-	<0.0001	Age
FI-Lab Q2 vs Q1	1.71 (0.90-3.28)	0.104		
FI-Lab Q3 vs Q1	2.58 (1.40-4.77)	0.002		
FI-Lab Q4 vs Q1	3.60 (1.98-6.53)	<0.0001		
Age (per year)	1.04 (1.01-1.07)	0.003		
Female sex	0.68 (0.47-0.99)	0.047		
FI-Lab (per 0.1 unit, continuous)	1.32 (1.18-1.49)	<0.0001		
**Model 3: M2 + BMI, Rx-Risk, insurance, residence [n=554, events=92]**				
FI-Lab Q1 (reference)	Reference	-	0.003	BMI
FI-Lab Q2 vs Q1	1.69 (0.86-3.30)	0.126		
FI-Lab Q3 vs Q1	2.35 (1.22-4.54)	0.011		
FI-Lab Q4 vs Q1	2.68 (1.38-5.19)	0.004		
Age (per year)	1.03 (1.00-1.06)	0.045		
Female sex	0.62 (0.40-0.96)	0.033		
BMI (per kg/m²)	0.97 (0.91-1.03)	0.287		
Rx-Risk score (unweighted)	0.99 (0.93-1.05)	0.641		
Medical aid	1.54 (0.91-2.60)	0.106		
Rural residence	1.02 (0.67-1.54)	0.943		
FI-Lab (per 0.1 unit, continuous)	1.25 (1.09-1.45)	0.002		
**Model 4: M3 + treatment delay [n=554, events=92]**				
FI-Lab Q1 (reference)	Reference	-	0.003	BMI
FI-Lab Q2 vs Q1	1.73 (0.88-3.38)	0.112		
FI-Lab Q3 vs Q1	2.46 (1.27-4.75)	0.008		
FI-Lab Q4 vs Q1	2.66 (1.37-5.16)	0.004		
Age (per year)	1.03 (1.00-1.06)	0.052		
Female sex	0.62 (0.40-0.97)	0.037		
BMI (per kg/m²)	0.97 (0.91-1.03)	0.273		
Rx-Risk score (unweighted)	1.01 (0.95-1.07)	0.874		
Medical aid	1.50 (0.89-2.54)	0.129		
Rural residence	1.00 (0.66-1.52)	0.987		
Treatment delay (per 14 days)	0.58 (0.30-1.13)	0.111		
FI-Lab (per 0.1 unit, continuous)	1.25 (1.08-1.44)	0.002		
**Model 5: M4 + corticosteroids, immunosuppressants, antidiabetic agents [n=554, events=92]**				
FI-Lab Q1 (reference)	Reference	-	0.004	Age, BMI
FI-Lab Q2 vs Q1	1.73 (0.88-3.39)	0.112		
FI-Lab Q3 vs Q1	2.39 (1.23-4.65)	0.010		
FI-Lab Q4 vs Q1	2.62 (1.35-5.10)	0.005		
Age (per year)	1.03 (1.00-1.07)	0.041		
Female sex	0.62 (0.39-0.96)	0.033		
BMI (per kg/m²)	0.97 (0.91-1.03)	0.281		
Rx-Risk score (unweighted)	1.00 (0.93-1.07)	0.889		
Medical aid	1.50 (0.88-2.54)	0.133		
Rural residence	1.02 (0.67-1.55)	0.938		
Treatment delay (per 14 days)	0.57 (0.29-1.13)	0.108		
Corticosteroid dose group	1.18 (0.78-1.79)	0.445		
Immunosuppressant use	1.19 (0.15-9.35)	0.866		
Antidiabetic agent use	0.98 (0.46-2.13)	0.968		
FI-Lab (per 0.1 unit, continuous)	1.25 (1.08-1.44)	0.003		

**Model 6: M5 + baseline cavitary disease [n=553, events=92]**				
FI-Lab Q1 (reference)	Reference	-	0.004	Age, BMI
FI-Lab Q2 vs Q1	1.71 (0.87-3.35)	0.121		
FI-Lab Q3 vs Q1	2.37 (1.22-4.61)	0.011		
FI-Lab Q4 vs Q1	2.60 (1.33-5.05)	0.005		
Age (per year)	1.03 (1.00-1.07)	0.037		
Female sex	0.62 (0.40-0.97)	0.035		
BMI (per kg/m²)	0.97 (0.91-1.03)	0.319		
Rx-Risk score (unweighted)	1.00 (0.93-1.07)	0.927		
Medical aid	1.46 (0.86-2.50)	0.162		
Rural residence	1.03 (0.67-1.57)	0.904		
Treatment delay (per 14 days)	0.58 (0.29-1.16)	0.126		
Corticosteroid dose group	1.18 (0.78-1.79)	0.443		
Immunosuppressant use	1.20 (0.15-9.34)	0.865		
Antidiabetic agent use	0.97 (0.45-2.09)	0.932		
Baseline cavitary disease	1.20 (0.70-2.06)	0.504		
FI-Lab (per 0.1 unit, continuous)	1.24 (1.08-1.44)	0.003		
**Model 7: M6 + baseline AFB smear positivity [n=485, events=83]**				
FI-Lab Q1 (reference)	Reference	-	0.0007	BMI
FI-Lab Q2 vs Q1	1.76 (0.85-3.64)	0.129		
FI-Lab Q3 vs Q1	2.89 (1.41-5.92)	0.004		
FI-Lab Q4 vs Q1	3.28 (1.60-6.73)	0.001		
Age (per year)	1.03 (1.00-1.07)	0.069		
Female sex	0.66 (0.41-1.07)	0.093		
BMI (per kg/m²)	0.99 (0.92-1.06)	0.739		
Rx-Risk score (unweighted)	1.00 (0.93-1.08)	0.922		
Medical aid	1.43 (0.81-2.54)	0.222		
Rural residence	1.13 (0.72-1.76)	0.592		
Treatment delay (per 14 days)	0.54 (0.26-1.12)	0.099		
Corticosteroid dose group	1.16 (0.74-1.80)	0.519		
Immunosuppressant use	1.10 (0.14-8.68)	0.929		
Antidiabetic agent use	0.95 (0.42-2.11)	0.890		
Baseline cavitary disease	1.17 (0.65-2.11)	0.597		
Baseline AFB smear positivity	0.65 (0.37-1.13)	0.126		
FI-Lab (per 0.1 unit, continuous)	1.31 (1.13–1.52)	0.0004		

[a] The column lists variables with significant violations (P<0.05)

Abbreviations: AFB, acid-fast bacilli, BMI, body mass index; CI, confidence interval; FI-Lab, laboratory-based frailty index; HR, hazard ratio; PH, proportional hazards

Supplementary Table S5: Competing Risks Analysis

Analysis/Model/Variable	csHR (95% CI)	P value	Observations, n	Events, n
**Death during treatment (competing event: discontinuation/success) [Log-rank Q1 vs Q4: p=0.006, overall: p=0.017]**				
**Model 1 [n=627, events=24]**				
FI-Lab Q1 (reference)	Reference	-		
FI-Lab Q2 vs Q1	1.18 (0.24-5.86)	0.838	627	24
FI-Lab Q3 vs Q1	3.03 (0.78-11.73)	0.109	627	24
FI-Lab Q4 vs Q1	4.89 (1.36-17.57)	0.015	627	24
P for trend	-	0.003		
**Treatment discontinuation (competing event: death) [Log-rank Q1 vs Q4: p=<0.0001, overall: p=0.0002]**				
**Model 1 [n=627, events=89]**				
FI-Lab Q1 (reference)	Reference	-		
FI-Lab Q2 vs Q1	2.25 (1.04-4.83)	0.038	627	89
FI-Lab Q3 vs Q1	3.18 (1.53-6.60)	0.002	627	89
FI-Lab Q4 vs Q1	4.29 (2.12-8.68)	0.0001	627	89
P for trend	-	<0.0001		
**Model 2 [n=627, events=89]**				
FI-Lab Q1 (reference)	Reference	-		
FI-Lab Q2 vs Q1	2.11 (0.98-4.54)	0.057	627	89
FI-Lab Q3 vs Q1	2.84 (1.36-5.92)	0.005	627	89
FI-Lab Q4 vs Q1	3.67 (1.80-7.51)	0.0004	627	89
Age (per year)	1.04 (1.01-1.07)	0.019	627	89
Female sex	0.70 (0.45-1.08)	0.106	627	89
P for trend	-	0.0002		
**Model 3 [n=554, events=66]**				
FI-Lab Q1 (reference)	Reference	-		
FI-Lab Q2 vs Q1	2.03 (0.92-4.45)	0.079	554	66
FI-Lab Q3 vs Q1	2.61 (1.21-5.64)	0.015	554	66
FI-Lab Q4 vs Q1	2.44 (1.10-5.38)	0.028	554	66
Age (per year)	1.03 (0.99-1.07)	0.157	554	66
Female sex	0.56 (0.33-0.96)	0.034	554	66
BMI (per kg/m²)	1.00 (0.93-1.08)	0.913	554	66
Rx-Risk score (unweighted)	0.97 (0.90-1.04)	0.337	554	66
Medical aid	2.24 (1.26-3.98)	0.006	554	66
Rural residence	0.91 (0.56-1.49)	0.710	554	66
P for trend	-	0.034		

Abbreviations: csHR, cause-specific hazard ratio; BMI, body mass index; CI, confidence interval; FI-Lab, laboratory-based frailty index

Supplementary Table S6: Net Reclassification Improvement (NRI) and Integrated Discrimination Improvement (IDI)[a]

	**Estimate (95% CI)[b]**	**P value**
**Reference: age + sex + Rx-Risk score (unweighted) → New: age + sex + Rx-Risk score (unweighted) + FI-Lab quartile (N = 627; events = 119[c]; non-events = 508[d])**		
**NRI (overall)**	0.342 (0.140–0.523)	0.001
NRI (events)	0.244	
NRI (non-events)	0.098	
**IDI**	0.026 (0.013–0.038)	0.001
**AUC: Reference = 0.627 → New model = 0.674 (ΔAUC = 0.047)**		

[a] MICE imputed dataset (m = 20, n = 627) used.
[b] 95% CI and P values derived from 1,000 bootstrap resamples.
[c] NRI events: proportion of cases with correct upward reclassification
[d] NRI non-events: proportion of non-cases with correct downward reclassification.
Abbreviations: AUC, area under the receiver operating characteristic curve; CI, confidence interval; IDI, integrated discrimination improvement; MICE, multiple imputation by chained equations; NRI, net reclassification improvement (category-free)(4)

Supplementary Table S7: Sensitivity Analyses

Analysis[a]	Model	N	Events	OR (95% CI)	P value	Notes
**Main analysis**	M2[b]	627	119	**3.63 (1.91-6.91)**	<0.0001	Complete case; reference row
(1) Alternative quartiles (>0.45 vs <0.10)	M2[b]	627	119	5.01 (2.30-10.90)	<0.0001	Quartiles by Howlett and colleagues
(2) Restriction to confirmed washout periods	M2[b]	582	111	4.37 (2.17-8.78)	<0.0001	Excludes patients with an index date before 2015-07-01 (n=45)
(3) Extended treatment success reclassified as failure	M2[b]	627	163	1.92 (1.14-3.25)	0.015	
(4) Narrower baseline laboratory window	M2[b]	603	116	3.62 (1.90-6.92)	0.0001	Uses the window of -30 to +7 days, instead of -60 to +7 days
(5a) Rx-Risk weighted, 365-day lookback	M3[c]	554	92	2.34 (1.16–4.73)	0.018	
(5b) Rx-Risk unweighted, 180-day lookback	M3[c]	554	92	2.38 (1.17–4.83)	0.017	
(6) Treatment success lower threshold ≥180 days	M2[b]	627	203	2.86 (1.72-4.75)	0.0001	
(7a) Non-inflammatory FI-Lab	M2[b]	626	119	2.99 (1.61-5.55)	0.0005	Excludes WBC, Neutrophil count, Lymphocyte count, Albumin, CRP
(7b) Non-inflammatory FI-Lab, continuous (per 0.1)	M2[b]	626	119	1.27 (1.10-1.47)	0.001	Excludes WBC, Neutrophil count, Lymphocyte count, Albumin, CRP
(7c) Non-inflammatory FI-Lab	M7[d]	485	83	2.48 (1.17–5.25)	0.018	Excludes WBC, Neutrophil count, Lymphocyte count, Albumin, CRP
(7d) Non-inflammatory FI-Lab, continuous (per 0.1)	M7[d]	485	83	1.24 (1.03–1.48)	0.020	Excludes WBC, Neutrophil count, Lymphocyte count, Albumin, CRP

[a] Highest vs. lowest FI-Lab quartile (Q4 vs. Q1) unless otherwise noted.
[b] Model 2: FI-Lab + age + sex
[c] Model 3: M2 + BMI, Rx-Risk, insurance, residence
[d] Model 7: fully adjusted
Abbreviations: BMI, body mass index; CI, confidence interval; CRP, C-reactive protein; FI-Lab, laboratory-based frailty index; OR, odds ratio; WBC, white blood cell count

Supplementary Table S8: Subgroup Analysis (FI-Lab Q4 vs Q1, Model 2[a])

Subgroup	N	Events	OR (95% CI)	P value
**Overall**	**627**	**119**	**3.63 (1.91-6.91)**	**<0.0001**
**Age group (P interaction[b] = 0.245)**				
65-74 yrs	235	34	2.74 (0.88-8.59)	0.083
≥75 yrs	392	85	4.04 (1.80-9.06)	0.0007
**Sex (P interaction[b] = 0.806)**				
Male	341	75	3.47 (1.51-7.99)	0.003
Female	286	44	3.66 (1.31-10.20)	0.013
**Baseline Cavitary disease (P interaction[b] = 0.141)**				
Absent	525	92	2.80 (1.40-5.61)	0.004
Present	101	27	13.23 (1.54-113.91)	0.019
**Baseline AFB smear status (P interaction[b] = 0.230)**				
Negative	396	77	3.20 (1.49-6.89)	0.003
Positive	148	29	12.65 (1.55-103.21)	0.018
**Corticosteroid use (P interaction[b] = 0.481)**				
No	583	109	3.68 (1.91-7.07)	<0.0001
Yes[c]	44	10	2.09 (0.06-73.57)	0.685
**Immunosuppressant use (P interaction[b] = 0.419)**				
No	620	117	3.57 (1.87-6.82)	0.0001
Yes[c]	7	2	NE	NE
**Antidiabetic agent use (P interaction[b] = 0.758)**				
No	557	104	3.70 (1.89-7.24)	0.0001
Yes	70	15	3.23 (0.30-34.58)	0.333
**Insurance type (P interaction[b] = 0.157)**				
National Health Insurance	548	100	3.91 (1.96-7.81)	0.0001
Medical aid	79	19	2.08 (0.35-12.42)	0.422
**Cavitary disease at 2 months (P interaction[b] = 0.423)**				
Resolved	47	8	3.52 (0.31-40.34)	0.312
Persisting[c]	18	7	NE	NE
**AFB smear status at two months (P interaction[b] = 0.138)[c]**				
Converted[c]	48	7	4.54 (0.21-99.03)	0.336
Still positive[c]	6	2	NE	NE

[a] Model 2: FI-Lab + age + sex

[b] P interaction: likelihood ratio test for multiplicative interaction.

[c] Firth penalized regression was used.

Abbreviations: AFB, acid-fast bacilli; CI, confidence interval; FI-Lab, laboratory-based frailty index; NE, not estimable (too few events per quartile for model convergence); OR, odds ratio

Supplementary Figure S1: Cumulative Incidence Functions – Competing Risks Analysis

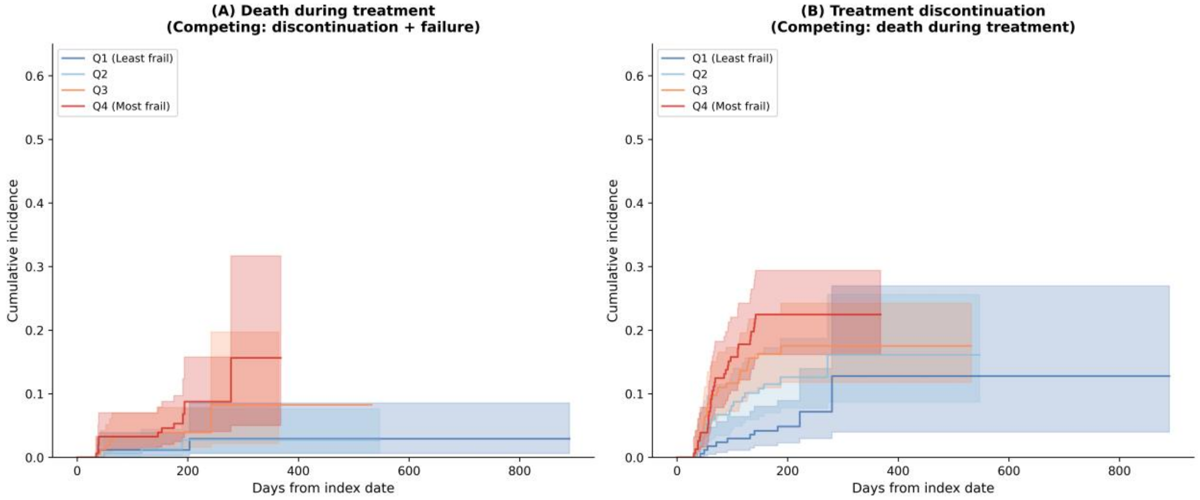

Cumulative incidence functions (Aalen-Johansen estimator) for death during treatment (**A**) and treatment discontinuation (**B**) by FI-Lab quartile, accounting for competing risks. For death during treatment, discontinuation and treatment success are treated as competing events; for discontinuation, death during treatment and treatment success are competing events. Abbreviations: FI-Lab, laboratory-based frailty index

Supplementary Figure S2: Calibration Plot

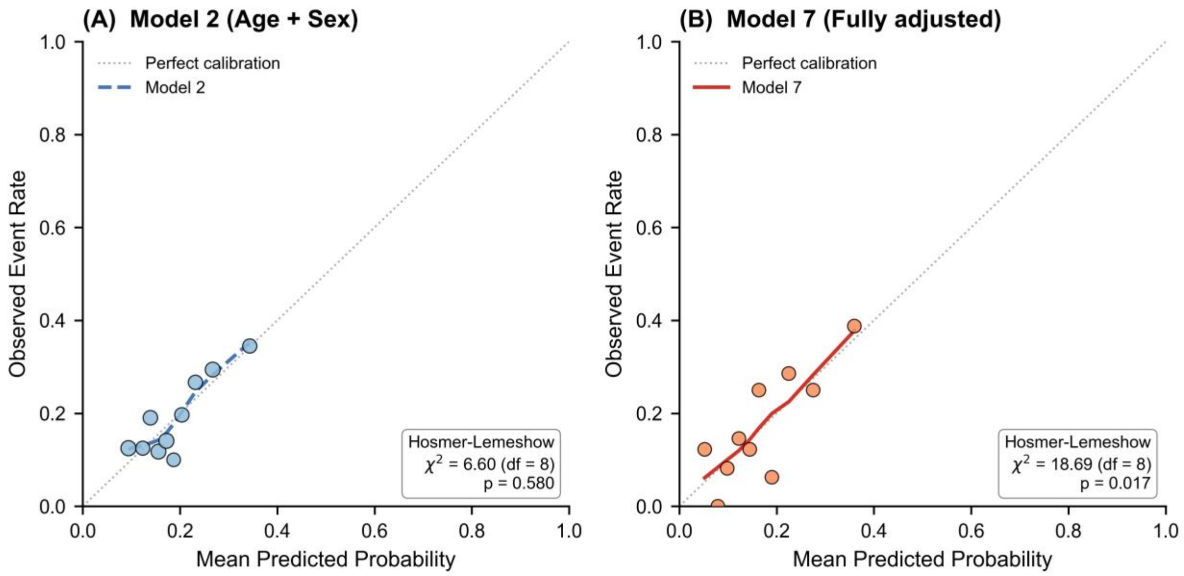

Calibration plot for the MICE Model 2 logistic regression (FI-Lab quartiles + age + sex). Observed event rates are plotted against mean predicted probabilities within decile groups. Circle size is proportional to the number of patients in each group. The dashed line represents perfect calibration. Hosmer–Lemeshow goodness-of-fit test result is displayed.
Abbreviations: FI-Lab, laboratory-based frailty index; MICE, multiple imputation by chained equations.

Supplementary Figure S3: Decision Curve Analysis – Net Benefit of FI-Lab-Augmented Model

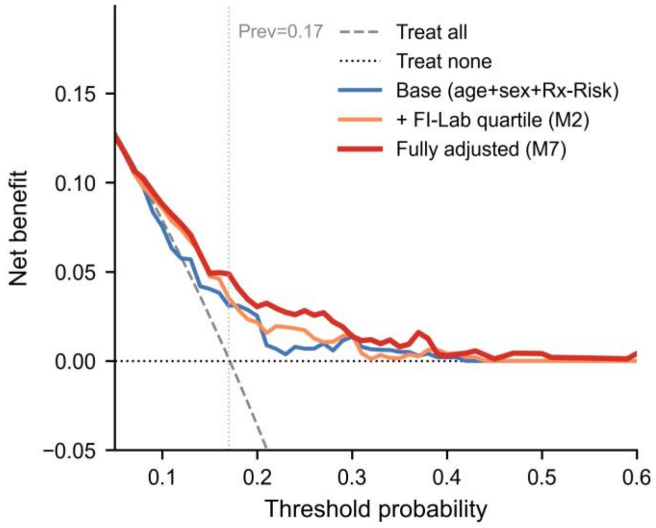

Decision curve analysis comparing the net benefit of three strategies across a range of threshold probabilities for the primary composite outcome: "treat all" (thin dashed), base model (age + sex + Rx-Risk; dashed), and FI-Lab-augmented model (Model 7; solid). Net benefit is defined as (true positive rate) − (false positive rate × threshold / (1 − threshold)). The FI-Lab-augmented model provides greater net benefit than the base model across clinically relevant threshold probabilities (approximately 10-40%), indicating potential utility in identifying high-risk patients for intensified monitoring or supportive interventions.

Abbreviations: FI-Lab, laboratory-based frailty index

STROBE Checklist – Cohort Study

Item No.	Recommendation	Reported on page/section
**Title & Abstract**		
1	Indicate the study design and state objectives	Title; Abstract
2	Provide an informative abstract (background, objectives, methods, results, conclusion)	Abstract
**Introduction**		
3	Explain the scientific background and rationale for the study	Introduction
4	State specific objectives, including pre-specified hypotheses	Introduction
**Methods**		
5	Study design: present key elements early in the paper	Materials and methods: Study design and setting
6	Setting: describe the setting, locations, and relevant dates (recruitment, exposure, follow-up, data collection)	Materials and methods: Study design and setting
7	Participants: cohort study — eligibility criteria, sources and methods of selection, follow-up methods	Materials and methods: Eligibility criteria; Figure 1
8	Variables: clearly define all outcomes, exposures, predictors, potential confounders, and effect modifiers	Materials and methods: Laboratory-based frailty index; Treatment outcomes; Covariates
9	Data sources/measurement: describe data sources and methods of assessment/measurement for each variable	Materials and methods: Laboratory-based frailty index; Covariates; Supplementary Table S1
10	Bias: describe any efforts to address potential sources of bias	Materials and methods: Statistical analysis; Discussion
11	Study size: explain how the study size was arrived at	Results: Cohort selection and baseline characteristics; Figure 1
12	Quantitative variables: explain how quantitative variables were handled; describe groupings	Materials and methods: Laboratory-based frailty index; Statistical analysis
13	Statistical methods: describe all statistical methods, including those used to control for confounding	Materials and methods: Statistical analysis; Supplementary Methods
14	Missing data: how missing data were addressed	Materials and methods: Statistical analysis; Supplementary Table S2
15	Cohort study: if applicable, explain how loss to follow-up was addressed	Materials and methods: Primary outcome; Supplementary Methods
16	Sensitivity analyses: report any sensitivity analyses performed	Materials and methods: Statistical analysis; Supplementary Table S7
**Results**		
17	Participants: report numbers at each stage of study; give reasons for non-participation at each stage	Results: Cohort selection and baseline characteristics; Figure 1
18	Descriptive data: characteristics of study participants; number of participants with missing data	Results: Cohort selection and baseline characteristics; Table 1; Supplementary Table S2
19	Outcome data: cohort study — report numbers of outcome events or summary measures over time	Results: Treatment outcomes; Table 2
20	Main results: give unadjusted estimates and, if applicable, confounder-adjusted estimates	Results: Treatment outcomes; Figure 3; Supplementary Tables S3-S4
21	Other analyses: report other analyses (subgroups, interactions, sensitivity)	Results: Discrimination, sensitivity, and subgroup analyses; Supplementary Tables S7-S8; Supplementary Figure S3
**Discussion**		
22	Key results: summarize key results with reference to study objectives	Discussion

23	Limitations: discuss limitations, taking into account sources of potential bias or imprecision	Discussion
24	Interpretation: give cautious overall interpretation of results; consider objectives, limitations, multiplicity	Discussion
25	Generalizability: discuss the generalizability (external validity) of the study results	Discussion
**Other**		
26	Funding: give the source of funding and the role of the funders	Funding statement

STROBE, Strengthening the Reporting of Observational Studies in Epidemiology

References

1. Howlett SE, Rockwood MRH, Mitnitski A, Rockwood K. Standard laboratory tests to identify older adults at increased risk of death. BMC Med. (2014) 12:171. doi: 10.1186/s12916-014-0171-9
2. Sapp DG, Cormier BM, Rockwood K, Howlett SE, Heinze SS. The frailty index based on laboratory test data as a tool to investigate the impact of frailty on health outcomes: a systematic review and meta-analysis. Age Ageing. (2023) 52(1):afac309. doi: 10.1093/ageing/afac309
3. Pratt NL, Kerr M, Barratt JD, Kemp-Casey A, Kalisch Ellett LM, Ramsay E, et al. The validity of the Rx-Risk Comorbidity Index using medicines mapped to the Anatomical Therapeutic Chemical (ATC) Classification System. BMJ Open. (2018) 8(4):e021122: doi: 10.1136/bmjopen-2017-021122
4. Pencina MJ, D'Agostino RB, D'Agostino RB, Vasan RS. Evaluating the added predictive ability of a new marker: from area under the ROC curve to reclassification and beyond. Stat Med. (2008) 27(2):157–172. doi: 10.1002/sim.2929
